# Autotoxicity of *Ambrosia artemisiifolia* and *Ambrosia trifida* and its significance for the regulation of intraspecific populations density

**DOI:** 10.1038/s41598-022-21344-8

**Published:** 2022-10-19

**Authors:** Pei Su, Xuelian Liu, Ruili Wang, Tong Liu, Wenxuan Zhao, Mingming Sun, Hanyue Wang, Yunxiao Liu, Qiang Wu

**Affiliations:** 1 Xinjiang Production and Construction Corps Key Laboratory of Oasis Town and Mountain-Basin System Ecology, Shihezi, 832003 Xinjiang China; 2 College of Life Sciences, Shihezi University, Shihezi, 832003 Xinjiang China; 3 Agricultural Technology Extension Station of Xinyuan County, Xinyuan, 835800 Xinjiang China; 4 Forestry and Grassland Work Station of Xinjiang Production and Construction Corps, Urumqi, 830011 Xinjiang China

**Keywords:** Ecology, Plant sciences

## Abstract

*Ambrosia artemisiifolia* and *Ambrosia trifida* are annual invasive plants that cause serious harm to agriculture, animal husbandry, and human health. Based on the important characteristic of high-density, cluster distribution of their populations, it is speculated that its autotoxins have an effect on density regulation. This study explored the regulation of autotoxicity on intraspecific density. We used water extracts from two plants to compare and verify the autotoxicity of seed germination, analysed the components of autotoxins. The results showed that *A. artemisiifolia* and *A. trifida* had significant autotoxicity, and the highest inhibition rates on seed germination were 27.21% and 77.94%, respectively; ultra-performance liquid chromatography tandem mass spectrometry analysis revealed that chlorogenic acid, caffeic acid, *p*-coumaric acid, and vanillin were the main autotoxins of the two plants. After the seeds were washed with water, the germination recovery rate of seeds increased with the increased of inhibition degree of autotoxins treatment. Therefore, this study verified the autotoxicity of *A. artemisiifolia* and *A. trifida*, which can promote and inhibit the seed germination of *A. artemisiifolia* and *A. trifida* to regulate intraspecific competition.

## Introduction

Allelopathy in plants affects the growth and development of the same or other species by releasing allelochemicals into the surrounding environment^1–3^. It plays a vital role in regulating intra- and inter-specific relationships^4^, changing microbial communities, and affecting plant growth and litter decomposition^5–7^. This affects plant community succession and formation^8,9^, and has an impact on ecosystem structure and function^10^. Allelopathy is also an important biological mechanism for invasive plants^11–13^.

*Ambrosia artemisiifolia* and *Ambrosia trifida* are plants that are common around the world^14,15^, they are native to North America, belong to the ragweed genus of the *Compositae* family, and are annual invasive weeds. They are characterised by having robust vigour, superior adaptability, high seed yields, and rapid dispersal^16^. In the past 200 years, *A. artemisiifolia* and *A. trifida* have spread to 80 and 40 countries, respectively^15^. Invasions by these weeds damage ecological environments, reduce biodiversity, and occupy farmland, thereby significantly reducing crop yields^14^. Furthermore, their pollen causes allergic dermatitis and bronchial asthma, which are harmful to human health^17,18^. Currently, these weeds are widely distributed in Northeast China, North China, Central China, and East China^19,20^. In 2010, these weeds were found in the Yili River Valley of China and were widely distributed along roadsides, as well as in farmland, pastures, and scenic spots. They have negatively affected the local ecological environment, and agricultural and animal husbandry production^21^. Both *A. artemisiifolia* and *A. trifida* are also resistant to some herbicides^22^, which certainly poses a major challenge for effective prevention and control measures. It is, therefore, necessary to increase our understanding of their invasive mechanisms from an ecological perspective to lay a foundation for efficient prevention and control.

The seed densities of *A. artemisiifolia* and *A. trifida* retained in the soil seed bank are extremely high. Typically, the yield per plant of the two species can reach up to 2000–62,000 seeds^23,24^, while the number of plants per unit area is 300–690 plants per square meter^25,26^. During a ragweed seed bank study, the estimated ragweed seed number in the upper 16 cm soil layer varied between 0 and 18 820 for a square meter in Hungary^27^. The seed bank per unit area is large, as only about 50% of the seeds are dispersed or eaten by insects^28,29^. Generally, after seed germination, high-density populations are formed that exhibit a strong self-thinning phenomenon^30^. To avoid intra-species competition, the two species undergo density regulation to avoid mass die-offs under high-density conditions; otherwise they would be eliminated by natural selection in high-density environments. Thus, regulation of seed germination is vital for buffering competition at the seedling stage.

Many studies have demonstrated that allelochemicals have self-poisoning effects, which reduce the population density by inhibiting seed germination and seedling growth of the same plant, thereby avoiding seedling competition for nutrients that subsequently affect adult plant growth and maintain population growth^31–34^. In terms of autotoxicity, the allelochemicals are also called autochemicals or phytotoxins^35^. At present, research on autotoxicity mostly focuses on continuous cropping obstacles and plantation decline^36–40^. However, the autotoxicity of invasive species has rarely been studied. Allelopathy is an important mode of invasion; while the allelopathic effects of *A. artemisiifolia* and *A. trifida* on companion species has been examined^41,42^, there are few studies on their autotoxicity. Whether the allelochemicals of *A. artemisiifolia* and *A. trifida* play a role in population density regulation is currently unclear.

Seed germination is a key link in the lifecycle of plants; it is crucial for the growth, development, and continuation of the population, and the community structure of plants^43–45^. The seed germination stage is the most critical stage for the use of allelochemicals^3^. In areas of intense intra-species competition, allelochemicals secreted by plant roots induce seed dormancy, thereby benefiting un-germinated seeds^46^. Using Petri dishes to investigate the effects of allelochemicals (or plant extracts) on seed germination is a valuable method in allelopathy response research^37,47–49^. Thus, we aimed to provide useful information on the effects of autotoxicity and invasion mechanisms through the effect of autotoxicity on seed germination. The goals of this study were to determine: (a) whether *A. artemisiifolia* and *A. trifida* present autotoxicity, (b) what is the specific composition of the autotoxins, and (c) what is the role of autotoxicity in *A. artemisiifolia* and *A. trifida* populations? Thus, the role of autotoxicity in protecting population stability and improving invasiveness can be analysed.

## Results

### Autotoxicity of *A. artemisiifolia* and *A. trifida* extracts

**Figure 1:**
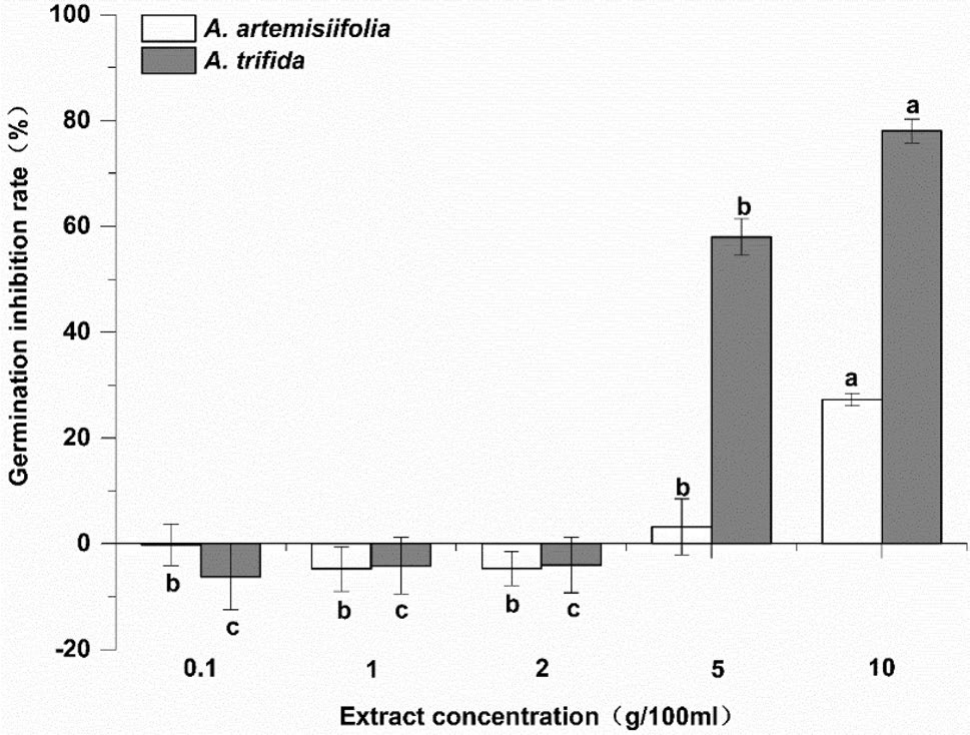
Autotoxicity of *A. artemisiifolia* and *A. trifida* extracts on their own seeds. Values are mean ± SE. Positive values indicate inhibited germination, and negative value indicate promoted germination. Different lowercase letters indicate that the seed germination inhibition rate of the same species at different concentrations was significantly different (*p* < 0.05). *A. artemisiifolia* seeds were treated with *A. artemisiifolia* plant extract, and *A. trifida* seeds were treated with *A. trifida* plant extract. EA.a: *A. artemisiifolia*; EA.t: *A. trifida*.

Autotoxicity was observed in both *A. artemisiifolia* and *A. trifida*. Low concentrations of their extracts promoted seed germination, whereas high concentrations significantly inhibited seed germination (Fig. 1). The results revealed that the germination of the two plants was promoted at 0.1, 1, and 2 g 100 mL^−1^ in their respective extracts, but significant differences were not detected. Further, the promotion rate was approximately 5%. The germination inhibition rate of *A. artemisiifolia* seeds was 3.22% at g 100 mL^−1^ and 27.21% at 10 g 100 mL^−1^. The germination inhibition rate of *A. trifida* seeds was 57.97% at 5 g 100 mL^−1^ and 77.94% at 10 g 100 mL^−1^. The autotoxic effects of *A. trifida* were much greater than those of *A. artemisiifolia* at 5 and 10 g 100 mL^−1^, based on seed germination performance*.*

**Figure 2:**
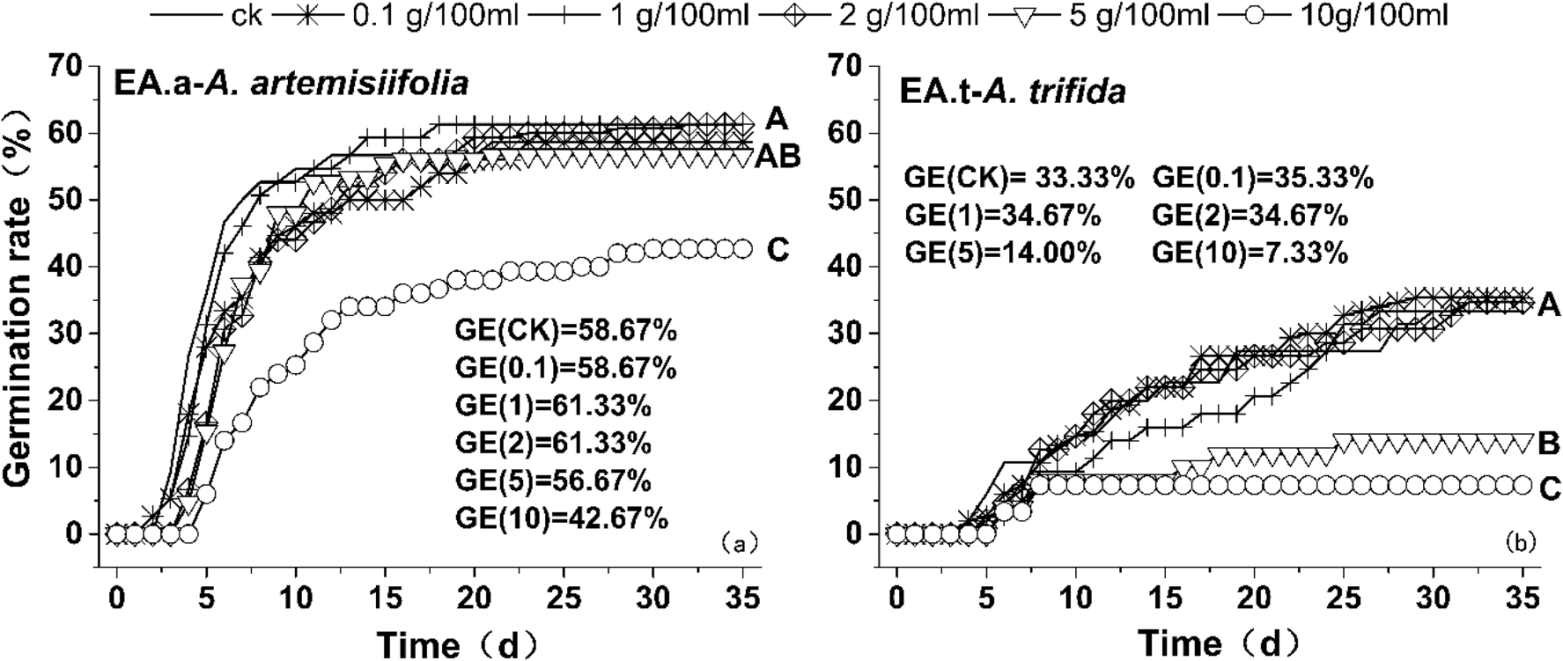
Germination potential of *A. artemisiifolia* and *A. trifida* on their own seeds. Different capital letters indicate significant difference in germination potential between concentration treatments (*p* < 0.05). The units of germination potential are g 100 mL.

Similarly, the autotoxic inhibition of the two species had a significant effect on germination potential only at high concentrations (Fig. 2). At 10 g 100 mL^−1^, the germination inhibition rate of *A. artemisiifolia* seeds was the highest and the germination time was the longest, starting after 5 d. The germination potential concentration (42.67%) was significantly lower than the other concentrations. The germination potential of the other treatment concentrations was approximately 60%, with no significant differences. *A. trifida* was strongly affected by autotoxicity inhibition and its seeds germinated slowly. At 5 and 10 g 100 mL^−1^, the germination potential of *A. trifida* seeds was significantly reduced (14% and 7.33%, respectively), and the initial germination time was delayed by 6 d. No significant differences were detected in the germination potential at other concentrations (~ 35%).

### Autotoxicity of each extract phase of *A. artemisiifolia* and *A. trifida* aqueous extracts

**Figure 3:**
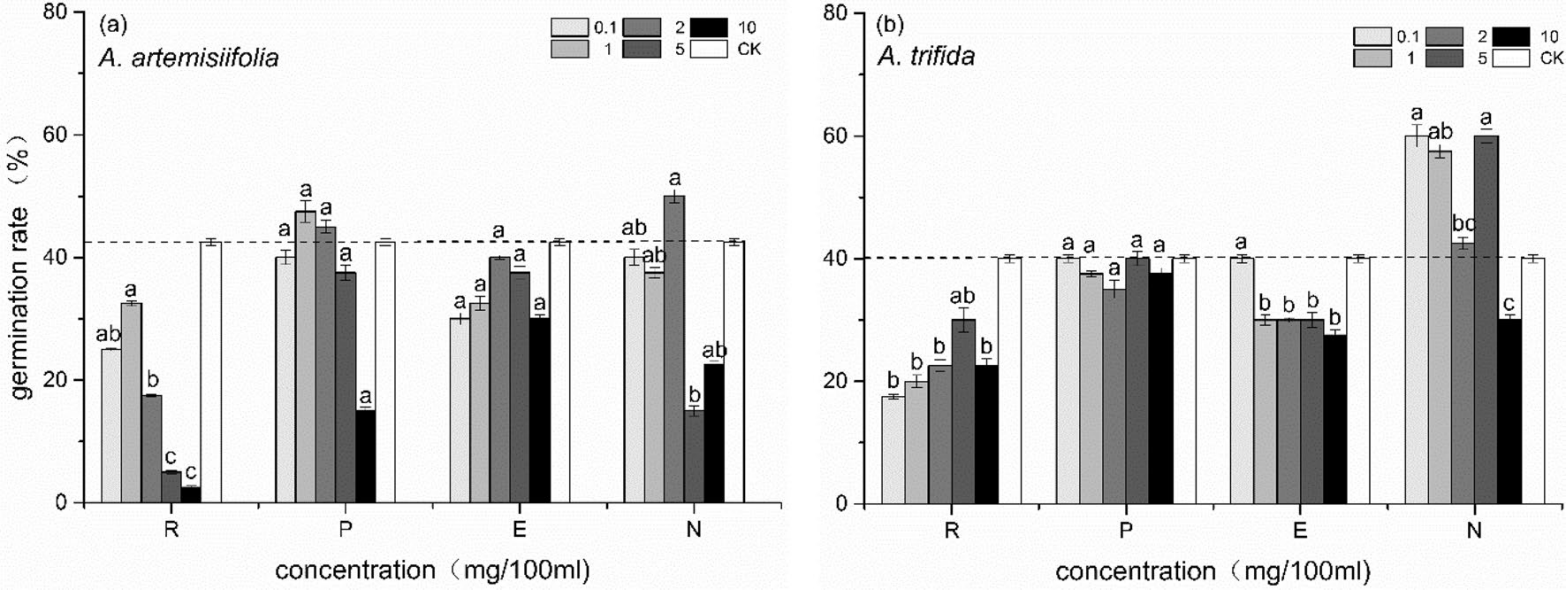
Effects of three organic extraction phases and residual water on seed germination rate of *A. artemisiifolia* (**a**) and *A. trifida* (**b**). In the figure, R represents the remaining water phase of the extraction, P represents the petroleum ether extraction phase, E represents the ethyl acetate extract phase and N represents the 1-butanol extract phase. Values are mean ± SE. Different lowercase letters indicate that the seed germination rate of the same species at different concentrations was significantly different (*p* < 0.05). *A. artemisiifolia* seeds were treated with *A. artemisiifolia* plant extract, and *A. trifida* seeds were treated with *A. trifida* plant extract. Dashed line shows the germination rate of control.

The petroleum ether, 1-butanol extract of *A. artemisiifolia,* and the 1-butanol extract of *A. trifida* promoted seed germination at low concentrations, and at high concentrations they inhibited seed germination (Fig. 3).

The concentration of petroleum ether extract solution which significantly promoted the germination of *A. artemisiifolia* seeds was 1 g 100 mL^−1^ (*p* < 0.05) and the promotion rate reached 12%. At a concentration of 10 g 100 mL^−1^, the germination of *A. artemisiifolia* seeds was significantly inhibited (*p* < 0.05) and the inhibition rate was 50%.

The concentration of the 1-butanol phase extract solution, which significantly inhibited the germination of *A. artemisiifolia* seeds, was 10 g 100 mL^−1^ (*p* < 0.05), with an inhibition rate of 47%. A concentration of 10 g 100 mL^−1^ significantly inhibited the seed germination of *A. trifida*, with an inhibition rate of 25%. The results showed that the autotoxic phases of *A. artemisiifolia* and *A. trifida* water extracts were petroleum ether phase, 1-butanol phase, and 1-butanol phase, respectively.

Using the UPLC-MS method, four autotoxins (vanillin, *p*-coumaric acid, caffeic acid, and chlorogenic acid) were identified in the petroleum ether phase and 1-butanol phase of *A. artemisiifolia* as well as in the 1-butanol phase of *A. trifida*. Table 1 shows the content of autotoxins and the germination promotion rate of their own seeds at different concentrations for *A. artemisiifolia* and *A. trifida*.

**Table 1:** Effects of different concentrations of water extracts of *A. artemisiifolia* and *A. trifida* on the contents of autotoxins and seed germination (*M* ± *SD*).

Species	Chemicals germination inhibition rate (%)	Plant water extract concentration (g 100 mL^−1^)				
		0.1	1	2	5	10
*A. artemisiifolia*	Vanillin (μg)	0.22	2.19	4.38	10.95	21.9
	Germination inhibition rate (%)	24 ± 0.27	33 ± 0.29	69 ± 0.27	18 ± 0.17	− 2 ± 0.06
	*P*-coumaric acid (μg)	0.09	0.93	1.86	4.65	9.3
	Germination inhibition rate (%)	11 ± 0.16	15 ± 0.28	20 ± 0.09	17 ± 0.23	9 ± 0.27
	Caffeic acid (μg)	0.71	7.12	14.24	35.6	71.2
	Germination inhibition rate (%)	10 ± 0.11	11 ± 0.24	15 ± 0.21	19 ± 0.21	20 ± 0.18
	Chlorogenic acid (μg)	26.32	263.22	526.44	1316.1	2632.2
	Germination inhibition rate (%)	38 ± 0.06	42 ± 0.23	24 ± 0.17	− 2 ± 0.13	− 38 ± 0.13
*A. trifida*	Vanillin (μg)	0.21	2.13	4.26	10.65	21.3
	Germination inhibition rate (%)	5 ± 0.19	2 ± 0.09	25 ± 0.16	33 ± 0.14	32 ± 0.14
	*P*-coumaric acid (μg)	0.11	1.09	2.18	5.45	10.9
	Germination inhibition rate (%)	0 ± 0	9 ± 0.14	11 ± 0.09	0 ± 0.24	− 7 ± 0.14
	Caffeic acid (μg)	0.20	1.98	3.96	9.9	19.8
	Germination inhibition rate (%)	− 6 ± 0.16	0 ± 0.16	11 ± 0.09	7 ± 0.08	− 11 ± 0.18
	Chlorogenic acid (μg)	7.53	75.26	150.52	376.3	752.6
	Germination inhibition rate (%)	17 ± 0.30	6 ± 0.23	39 ± 0.21	− 17 ± 0.11	− 24 ± 0.19

Positive value of germination inhibition rate (%) means promoting germination, negative value means inhibiting germination.

As shown in Table 1, the content of autotoxins per unit mass of *A. artemisiifolia* plants was higher than that of *A. trifida*. When treated separately with the four substances, the treatments of *A. artemisiifolia* and *A. trifida* seeds had certain effects on the seed germination of the two species.

Among them, vanillin promoted the germination rate of *A. artemisiifolia* seeds by 69% and 33% for *A. trifida.* The germination inhibition rate of chlorogenic acid in *A. artemisiifolia* seeds was 38%, and that of *A. trifida* was 24%. Moreover, the effect of each substance on the seed germination of *A. artemisiifolia* was greater than that of *A. trifida*.

Studies have found that the chlorogenic acid content in *A. artemisiifolia* and *A. trifida* is 263.22 μg g^−1^ and 75.26 μg g^−1^, respectively, which are much higher than those of the other three substances. Therefore, a higher concentration of chlorogenic acid is required to achieve the effect of inhibiting seed germination.

Although *p*-coumaric acid and caffeic acid have certain allelopathic and autotoxic effects on *A. artemisiifolia* and *A. trifida*, they are not as effective as chlorogenic acid and vanillin on the two species. Therefore, it is believed that these four substances are autotoxins in *A. artemisiifolia* and *A. trifida*, but chlorogenic acid and vanillin play a more important role.

As shown in Table 2, the concentration treatments showed promoting and inhibiting effects, which verified that chlorogenic acid and vanillin are autotoxins of *A. artemisiifolia* and *A. trifida*.

**Table 2:** Verification effects of chlorogenic acid and vanillin on seed germination of *A. artemisiifolia* and *A. trifida* (*M* ± *SD*).

Species	Chemicals germination inhibition rate (%)	Concentration (μg 100 mL^−1^)					
*A. artemisiifolia*	Chlorogenic acid (μg)	25	100	500	1500	3000	
	Germination inhibition rate (%)	38 ± 0.06	42 ± 0.23	24 ± 0.17	− 2 ± 0.23	− 38 ± 0.13	
	Vanillin (μg)	0.2	2	4	20	1000	2000
	Germination inhibition rate (%)	24 ± 0.27	33 ± 0.29	69 ± 0.27	− 2 ± 0.06	− 16 ± 0.17	− 16 ± 0.25
*A. trifida*	Chlorogenic acid (μg)	10	50	100	500	1000	
	Germination inhibition rate (%)	17 ± 0.30	6 ± 0.23	40 ± 0.21	− 17 ± 0.11	− 24 ± 0.19	
	Vanillin (μg)	0.2	2	4	20	1000	2000
	Germination inhibition rate (%)	6 ± 0.19	2 ± 0.09	25 ± 0.16	32 ± 0.14	6 ± 0.23	− 17 ± 0.14

Positive value of germination inhibition rate (%) means promoting germination, negative value means inhibiting germination.

In *A. artemisiifolia*, chlorogenic acid and vanillin promoted seed germination at 100 μg 100 mL^−1^ and 4 μg 100 mL^−1^, and the germination promotion rates were 42% and 69%, respectively. Seed germination was inhibited at 1500 μg 100 mL^−1^ and 20 μg 100 mL^−1^, and the germination inhibition rate was 2%.

In *A. trifida*, chlorogenic acid and vanillin promoted seed germination at 100 μg 100 mL^−1^ and 20 μg 100 mL^−1^, and the germination promotion rates were 40% and 32%, respectively. At 500 μg 100 mL^−1^ and 2000 μg 100 mL^−1^ chlorogenic acid and vanillin inhibited seed germination, and the germination inhibition rate was 17%.

### Vitality recovery of un-germinated seeds

**Figure 4:**
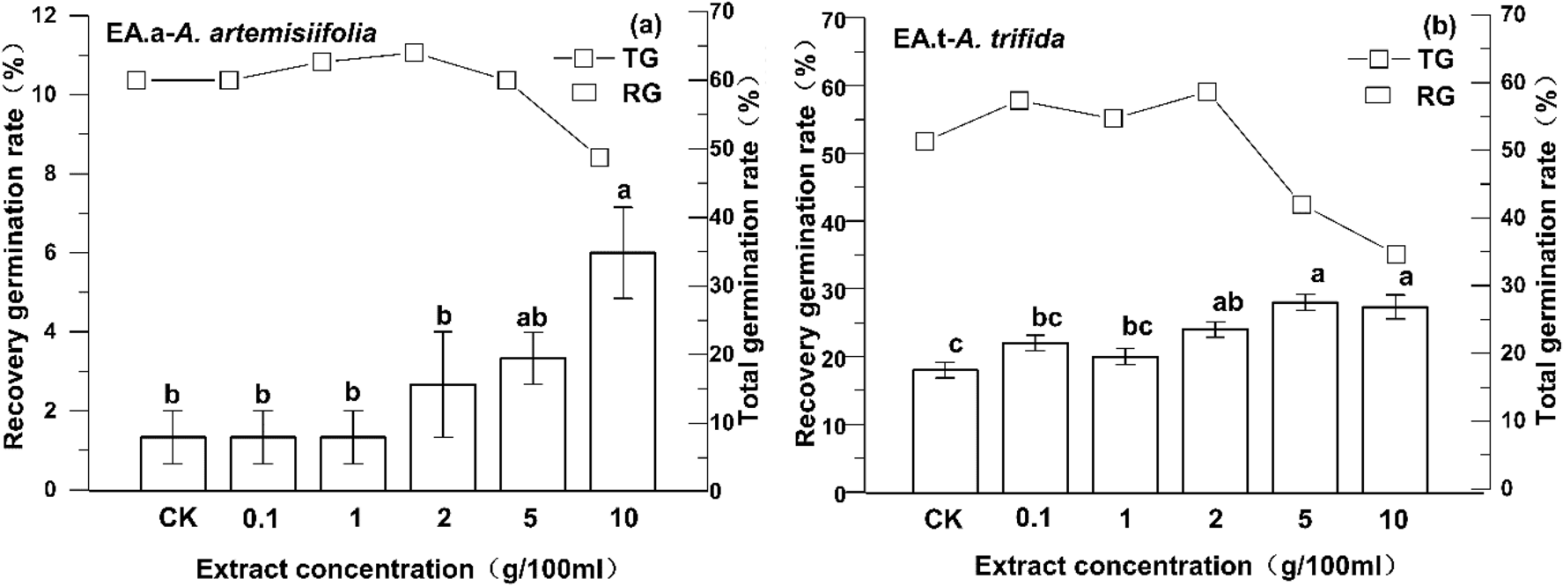
Recovery germination rate and total germination rate of non-germinated seeds (**a** *A. artemisiifolia*, **b** *A. trifida*). Lowercase letters indicate that significant differences in the recovery germination rate of non-germinated seeds at different concentrations (*p* < 0.05).

A positive correlation was detected between recovery germination rate and the concentrations of the two sets of extracts (Fig. 4). The higher the extract concentration, the higher the seed recovery germination rate was. The recovery germination rate of *A. artemisiifolia* extracts treated with 10 g 100 mL^−1^ was different from those treated with 5 g 100 mL^−1^; this difference was not significant but was significantly higher than that of the other concentrations. The recovery germination rate of *A. trifida* extracts treated with 5 and 10 g 100 mL^−1^ were significantly higher than that of the control, and at 0.1 and 1 g 100 mL^−1^, but the difference was not significant compared to 2 g 100 mL^−1^.

Initially, the total germination rate of the two species increased, but then decreased as the concentration increased (Fig. 4). Seeds treated with the two extracts at 2 g 100 mL^−1^ had the strongest recovery after washing. The total germination rate of both species treated with 0.1, 1, and 2 g 100 mL^−1^ was higher than that of the control. That is, the total germination rate of both species after treatment with low-concentration extracts improved compared to the control. Additionally, the total germination rate under 5 and 10 g 100 mL^−1^ was lower than that of other concentrations; in other words, high concentrations negatively affected seed total germination rate.

## Discussion

### *A. artemisiifolia* and *A. trifida* have significant autotoxicity; low concentrations promote seed germination and high concentrations inhibit seed germination

A large number of studies have shown that allelopathy is an important invasion mechanism for many alien species^50–52^. *A. artemisiifolia* and *A. trifida* have strong allelopathic effects^41^, but there are few studies on the autotoxicity of these two invasive plants. This study verified the autotoxicity of *A. artemisiifolia* and *A. trifida*, and determined its effective concentrations.

In our experiment, the autotoxins of *A. artemisiifolia* and *A. trifida* significantly inhibited the germination of their own seeds. They promoted the germination of their own seeds at lower concentrations, and at the same time promoted the germination rate and germination potential. The same effect was observed when treated with separate autotoxins (chlorogenic acid and vanillin). The plant water extracts promoted seed germination at low concentrations and accelerated the germination speed 2 days and 4 days earlier, respectively, and the effect of treatment with autotoxic substances was more significant 4 days and 6 days earlier, respectively.

High concentrations of autotoxins inhibition seed germination; as the concentration of the extract increased, the germination inhibition rate was greater. At the same time, the germination rate and germination vigour of *A. artemisiifolia* and *A. trifida* seeds was reduced. The plant water extract inhibited seed germination at high concentrations and delayed the germination speed by 5 days and 6 days, respectively, and treatment with autotoxic substances was delayed by 2 days and 3 days, respectively. This low concentration promoted seed germination and the high concentrations inhibited seed germination. These findings are consistent with the "mass concentration effect" reported in most studies on allelopathy and autotoxicity^53^.

Kumari and Kohli^54^ found that the water extract of *A. artemisiifolia* leaves, at a concentration of 0.5 g mL^−1^, had a lethal rate of 50% to its own plants. Our study found that the aqueous extracts of *A. artemisiifolia* and *A. trifida* plants have a strong inhibitory effect on the germination of their own seeds, demonstrating an inhibitory rate for *A. artemisiifolia* of up to 27.21% at a concentration of 0.1 g mL^−1^. Also found that *A. trifida* has an inhibition rate of 77.94% on its own seed germination at a concentration of 0.1 g mL^−1^. Both proved that *A. artemisiifolia* and *A. trifida* have strong autotoxicity.

### Chlorogenic acid, caffeic acid, *p*-coumaric acid, and vanillin are the main autotoxic substances in *A. artemisiifolia *and *A. trifida*, among which chlorogenic acid and vanillin have the most significant effects

There are many types of autotoxins. Common compounds can be divided into 10 categories according to their structure and properties: simple water-soluble organic acids, linearalcohols, aliphatic aldehydes, and ketones; simple unsaturated lactones; long-chain fatty acids and polyalkynes; benzoquinones, anthraquinones, and double quinones; simple phenols, benzoic acid, and their derivatives; cinnamic acids and their derivatives; coumarins; flavonoids; tannins; and terpenoids, enes, and zirconias^55–57^.

We used UPLC-MS to detect and analyse compounds from *A. artemisiifolia* and *A. trifida* and found that chlorogenic acid, caffeic acid, vanillin, and *p*-coumaric acid were the main autotoxins of the two species. The types of autotoxins in the two species were the same. However, the effect of the autotoxins on *A. artemisiifolia* was greater than that of *A. trifida*. Except for *p*-coumaric acid, the content of various substances in *A. artemisiifolia* was higher than that in *A. trifida*. For example, the content of chlorogenic acid (263.22 μg g^−1^) in *A. artemisiifolia* was close to four times that of *A. trifida* (75.2 μg g^−1^).

Further investigation found that chlorogenic acid and vanillin had the most apparent autotoxicity effects on *A. artemisiifolia* and *A. trifida*. The content of vanillin in *A. artemisiifolia* and *A. trifida* was similar, but *A. artemisiifolia* was more sensitive to the effects of vanillin, and a higher concentration was required in *A. trifida*. The autotoxicity effect of vanillin on *A. artemisiifolia* and *A. trifida* was demonstrated for the first time in this study.

Caffeic acid and *p*-coumaric acid have been reported in *A. artemisiifolia* and *A. trifida*. These have inhibitory effects on the seed germination and seedling growth of *Digitaria ciliaris*, *Echinochloa crus-galli*, and *Cyperus microiria*^58^. In this study, we found that there is a certain degree of autotoxicity on the seeds of *A. artemisiifolia* and *A. trifida*, but the effect on seed germination was not significant.

### Autotoxicity of *A. artemisiifolia* and *A. trifida* is an important means of population density regulation

Generally speaking, under natural conditions, the allelochemicals produced by plants can enter the surrounding environment in the following ways: (1) the aerial parts of plants release volatile substances into the environment, (2) the leaching effect of rain and mist, (3) plant root exudates are secreted into the soil rhizosphere, and (4) when plant tissue litter and plant debris are decomposed, especially the decomposition of residual roots. Most plant allelochemicals enter the surrounding environment through these methods to affect the growth of the plant itself or the surrounding plants, and the longer the growth and distribution of the species, the greater the accumulation of autotoxins.

For perennial plants, it is logical to use autotoxicity to avoid sibling competition, but for annual plants, the longer a population is established, the more autotoxins accumulate. This inhibits their own seed germination and plant growth, does not prevent sibling competition, and promotes population increase. Friedman et al.^59^ found that the autotoxicity of annual plants can cause seeds to germinate or spread further from the parent, delay their germination, or germinate only after rainfall. From their study of the autotoxicity of *Lolium rigidum*, Canals et al.^60^ believed that the autotoxicity of annual plants improves the viability of the population by inhibiting the development of populations with fewer individuals, and chemical interference and resource competition are closely related processes. Emeterio^61^ believes that chemical substances have a significant density-dependent effect on plant growth. Autotoxicity may act as a self-regulator of species population density to prevent intra-specific competition^44^. However, there are few reports on the effects of autotoxicity on the populations of annual invasive plants.

*Ambrosia artemisiifolia* and *A. trifida* are significantly harmful, invasive plants globally, and their invasion mechanisms are a focus for current research efforts. The two species’ short distance dispersal methods and high-density cluster distributions may be an important feature that strengthens resource competition and enables invasive success^28,62,63^. Understanding the regulation of intra-species density at high densities is an interesting scientific challenge. Our research found that the autotoxins of the two species promote their own seed germination effects at low concentrations. Therefore, in the early stage of colonisation by both species, the population density and the concentration of autotoxins are low at this time, and this promotes seed germination and improved competitiveness, thereby gaining an inter-specific competitive advantage for the community. However, when the population has been continuously growing for many years, the population becomes densely distributed and as the intra-specific individual numbers increase, autotoxins continuously accumulate. When the self-inhibition concentration is reached, the promoting effect on seed germination is changed to an inhibitory effect, which effectively prevents population over-density. This is a common feature of plant autotoxicity^64–66^. The recovery of seed germination rates after washing with water further proved autotoxicity. In conclusion, the autotoxicity of *A. artemisiifolia* and *A. trifida* is an important means by which these populations regulate density and alleviate intra-specific competition during seed germination.

## Conclusions

Based on seed bioassay method and UPLC-MS analysis technology, the autotoxicity of *A. artemisiifolia* and *A. trifida* and the main components of autotoxics were studied. The results showed that there was autotoxicity in both plants, and the main autotoxics were chlorogenic acid, caffeic acid, *p*-coumarin acid and vanillin. Among them, these four substances have promoting and inhibiting concentrations on the seeds of *A. artemisiifolia* and *A. trifida* under indoor test conditions, but their practical effects under natural conditions needs to be further studied.

## Materials and methods

### Experimental material selection and origin

Plants and seeds of *A. artemisiifolia* and *A. trifida* were collected from areas that had been invaded by these species in Xinyuan County, China. The plant material used in this study was collected under the permission from the Xinyuan County and followed the established rules and regulations. Experimental research and field studies on plants, including the collection of plant material, complied with relevant institutional, national, and international guidelines and legislation.

In September 2018, 300 plants each of *A. artemisiifolia* and *A. trifida* were randomly collected from the sampling of site, dried under natural conditions, and collected the seeds. According to the plant segments divided by Liu et al.^67^, the seeds on the top of the plants were selected as the research objects. The seeds were stored in a kraft paper bag at room temperature, wherein the after-ripening time of *A. artemisiifolia* seeds was 42 days, and the after-ripening time of ragweed *A. trifida* seeds was 45 days.

In October 2018, 300 complete plants each of mature *A. artemisiifolia* and *A. trifida* were randomly collected from the plot. The voucher specimens of *A. artemisiifolia* and *A. trifida* are stored in the Herbarium of Shihezi University (Xingjiang, China), and the numbers of specimen were No.11265 and No.11272 respectively.

## Experimental design

### Extract preparation

The concentrations used in this study were based on a preliminary experiment. In the preliminary experiment, half of the seeds treated with 2 g 100^−1^ mL extracts germinated successfully. To determine the concentration that completely inhibited seed germination, the highest concentration of the extract was set to 10 g 100^−1^ mL. This paper primarily studied the effect of autotoxin dosage, which does not need to be exactly the same as that found in natural habitats. The collected whole plants (including the root system) of both species were dried in an electric constant temperature drying box at 120 °C, then separately crushed and thoroughly mixed at room temperature to eliminate the differences between plants. All experiments in the article requiring the use of plant extracts were prepared using the whole plant (including the aerial and underground parts of the plant). And in order to ensure consistent solubility of the treatment solutions, all treatment solutions were prepared the day before the experiment.

Samples (0.1, 1, 2, 5, and 10 g) were soaked in 100 mL distilled water and filtered after 36 h. Samples were centrifuged at 3000 r min^−1^ for 10 min, and extracts were prepared at concentrations of 0.1, 1, 2, 5, and 10 g 100^−1^ mL. The 10 treatment solutions were stored in a refrigerator at 4 °C.

### Preparation of organic extraction phase of aqueous extracts of *A. artemisiifolia* and *A. trifida*

Using the same method as above, 100 g of the two crushed plant samples were placed into 1 L of distilled water to prepare the water extracts.

The water extract was successively extracted with petroleum ether, ethyl acetate, and 1-butanol. Ten millilitres of each phase was reserved for qualitative and quantitative analyses. The rest was concentrated on a rotary evaporator to obtain the petroleum ether phase (*A. artemisiifolia* 16.71 mg g^−1^, *A. trifida* 32 mg g^−1^), ethyl acetate phase (*A. artemisiifolia* 44.78 mg g^−1^, *A. trifida* 33 mg g^−1^), 1-butanol phase (*A. artemisiifolia* 18 mg g^−1^, *A. trifida* 24.9 mg g^−1^), and the remaining water phase (*A. artemisiifolia* 34.6 mg g^−1^, *A. trifida* 41 mg g^−1^). The four dry substances were redissolved in distilled water to prepare the plant concentrations corresponding to the preliminary experiment. The 40 treatment solutions were stored in a refrigerator at 4 °C for the seed germination experiments.

### Identification and analysis of the extraction phase of autotoxins

The 10 ml of extraction liquid reserved in the previous experiment was blow-dried withN_2_. The dry matter was dissolved in 1 mL methanol and centrifuged at 12,000 r min^−1^ for 10 min, and the supernatant was collected and filtered through a 0.22 μm membrane.

For the ultra-performance liquid chromatography tandem mass spectrometry (UPLC-MS) method, the instruments used were an ACQUITY UPLC ultra-high performance liquid chromatograph, a XEVO TQ-S triple quadrupole tandem mass spectrometer, and a MassLynx workstation (Waters company, USA; Waters ACQUITY UPLC BEH C18 column (50 mm × 2.1 mm, 1.7 μm)). The conditions were as follows: flow rate at 0.3 mL min; injection volume of 1 μL; and column oven at 30 °C. For the mass spectrometry conditions, the ion source was an electrospray ionisation source and the multi-reaction detection mode (MRM) was used for content determination. The desolventising gas temperature was 450 °C, source temperature was 150 °C, desolventising gas flow rate was 800 L h^−1^, cone gas was 150 L h^−1^, and capillary voltage was 3000 V.

The gradient elution procedures of mobile phase A (0.1% formic acid–water) and B (acetonitrile) are shown in Table 3.

**Table 3:** Mobile phase gradient elution procedure.

Time (min)	Flow rate (mL min^−1^)	0.1% formic acid–water (%)	Acetonitrile (%)
0	0.3	86	14
2.0	0.3	86	14
3.0	0.3	5	95
3.5	0.3	5	95
4.0	0.3	86	14
6.0	0.3	86	14

Using the external standard method, solutions were prepared containing eight standard substances (all chromatographically pure) containing chlorogenic acid, borneol, acetyl borneol, caffeic acid, cinnamyl alcohol, *p*-coumaric acid, azelaic acid, and vanillin. The concentrations were 1, 10, 25, 50, 100, 500, and 1000 μg 10 mL^−1^. According to the determined chromatographic conditions, standard solutions of different concentrations were used for analysis and a standard curve was drawn. Qualitative and quantitative analyses of the samples were performed. Through this process, four chemical substances were identified and quantified: chlorogenic acid (*A. artemisiifolia* 263.22 μg g^−1^, *A. trifida* 75.26 μg g^−1^), caffeic acid (*A. artemisiifolia* 7.12 μg g^−1^, *A. trifida* 1.98 μg g^−1^), *p*-coumaric acid (*A. artemisiifolia* 0.93 μg g^−1^, *A. trifida* 1.09 μg g^−1^), and vanillin (*A. artemisiifolia* 2.19 μg g^−1^, *A. trifida* 2.13 μg g^−1^) (see Supplementary Fig. S2).

### Seed germination experiment

The plant extracts and autotoxic substances of *A. artemisiifolia* and *A. trifida* were used to treat their own seeds. The experiment was divided into three parts.

First, the seeds were treated directly with the plant water extracts of *A. artemisiifolia* and *A. trifida*. To determine the effect of different plant extract concentrations on seed germination, we set the concentrations at 0.1, 1, 2, 5, 10 g 100 mL^−1^, for a total of 10 treatments.

Second, to determine the specific chemical substances, the water extracts of the plants were extracted, and the dry substances of each extract phase were formulated into the corresponding concentration according to the plant concentration. This yielded a total of 40 treatments.

Third, to determine the specific action concentrations of the autotoxins of *A. artemisiifolia* and *A. trifida*, two sets of experimental concentrations were set up. The corresponding concentrations of autotoxins (chlorogenic acid, caffeic acid, *p*-coumaric acid, and vanillin) were set according to the plant concentrations, at five concentrations for each substance, yielding a total of 40 treatments. To verify the germination effect of chlorogenic acid and vanillin on *A. artemisiifolia* and *A. trifida* seeds, five concentrations of chlorogenic acid and six concentrations of vanillin were set, with a total of 22 treatments (Table 4).

**Table 4:** Specific concentration Settings of chlorogenic acid and vanillin verification experiments.

Species	Chemicals	Concentrations (μg 100 mL^−1^)					
*A. artemisiifolia*	Chlorogenic acid	25	100	500	1500	3000	
	Vanillin	0.2	2	4	20	1000	2000
*A. trifida*	Chlorogenic acid	10	50	100	500	1000	
	Vanillin	0.2	2	4	20	1000	2000

Experiments were carried out in Petri dishes (90 mm diameter) containing a double layer of filter paper with 25 seeds of one species evenly spread across each dish. The Petri dishes were then placed in a light growth incubator under a 12/12 h light/dark photoperiod, with a light intensity of 3000 lx at 25/15 °C to simulate day/night temperatures of the species’ original habitat from April to May. Distilled water was used as the control (control). Each factor combination was replicated thrice.

Seed germination was defined as the emergence of the radicle. The number of germinated seeds was recorded daily and a small amount of distilled water was added to keep the double-layer filter paper moist for 5 weeks. Germination and germination inhibition rates were calculated as follows:

1 $${\text{Germination}}\,{\text{rate}} = {\text{number}}\,{\text{of}}\,{\text{normal}}\,{\text{germinated}}\,{\text{seeds}} \cdot {\text{number}}\,{\text{of}}\,{\text{tested}}\,{\text{seeds}} \times {1}00\,(\% )$$

2 $${\text{Germination}}\,{\text{inhibition}}\,{\text{rate}} = ({\text{germination}}\,{\text{rate}}\,{\text{of}}\,{\text{control}} - {\text{germination}}\,{\text{rate}}\,{\text{of}}\,{\text{the}}\,{\text{concentration}}\,{\text{treatment}}) \times {\text{germination}}\,{\text{rate}}\,{\text{of}}\,{\text{control}} \times {1}00\,(\% )$$

Positive values indicated inhibited germination, whereas negative values indicated promote germination. Germination potential was calculated as follows:

3 $${\text{Germination}}\,{\text{potential}} = {\text{cumulative}}\,{\text{number}}\,{\text{of}}\,{\text{germinated}}\,{\text{seeds}}\,{\text{within}}\,{\text{a}}\,{\text{specified}}\,{\text{time}} \cdot {\text{number}}\,{\text{of}}\,{\text{tested}}\,{\text{seeds}} \times {1}00\,(\% )$$

The germination period of both *A. artemisiifolia* and *A. trifida* was 35 days.

### Seed vitality restoration test

To study the effects of allelochemicals on *A. artemisiifolia* and *A. trifida* seed vigour, restoration experiments were conducted. Seeds that did not germinate after culturing in the two types of extracts for five weeks were washed in distilled water and cultured in new Petri dishes with clean distilled water at 15–25 °C. The recovery germination rate and total germination rate were calculated as follows:

4 $${\text{Recovery}}\,{\text{germination}}\,{\text{rate}} = {\text{number}}\,{\text{of}}\,{\text{germinated}}\,{\text{seeds}}\,{\text{after}}\,{\text{wash}}\,{\text{treatment}} \cdot {\text{number}}\,{\text{of}}\,{\text{total}}\,{\text{seeds}}\,{\text{tested}} \times {1}00\,(\% )$$

5 $${\text{Total}}\,{\text{germination}}\,{\text{rate}} = \left( {{\text{number}}\,{\text{of}}\,{\text{germinated}}\,{\text{seeds}}\,{\text{treated}}\,{\text{with}}\,{\text{extract}} + {\text{number}}\,{\text{of}}\,{\text{germinated}}\,{\text{washed}}\,{\text{seeds}}} \right) \cdot {\text{number}}\,{\text{of}}\,{\text{total}}\,{\text{seeds}}\,{\text{tested}} \times {1}00\,(\% )$$

### Data analysis

Using concentration as the test factor, and germination inhibition rate as a response variable, the seed responses at different concentrations were analysed by one-way ANOVA (*p* < 0.05). When the significant difference was greater than 0.05, the null hypothesis was accepted and the variance was considered to be homogeneous. Duncan’s multiple range test was used to test for significant differences in the inhibition rate and germination potential between treatments with different concentrations. The germination responses of different seed sizes were analysed using an independent-sample t-test (*p* < 0.05). Seed size was used as a test factor and germination rate was the response variable. All analyses were performed using SPSS v19.0 (SPSS, Chicago, IL, USA) for Windows. All figures were constructed using Origin v9.0 (OriginLab, Northampton, MA, USA) for Windows.

## Supplementary Information

Supplementary Information.

## Data Availability

The data that supports the findings of this study are available in the supplementary material of this article.
