# Supplementary material for: Autotoxicity of Ambrosia artemisiifolia and Ambrosia trifida and its significance for the regulation of intraspecific populations density
Source: Sci Rep. 2022 Oct 19;12:17424. doi: 10.1038/s41598-022-21344-8 (PMC9582198; doi:10.1038/s41598-022-21344-8)
Supplement: Supplementary file 1 — Supplementary Information. [file 41598_2022_21344_MOESM1_ESM.docx]

**Supplementary Fig. S2. UPLC-MS analysis results of A. artemisiifolia and A. trifida**

**1. Standard Curve**

**
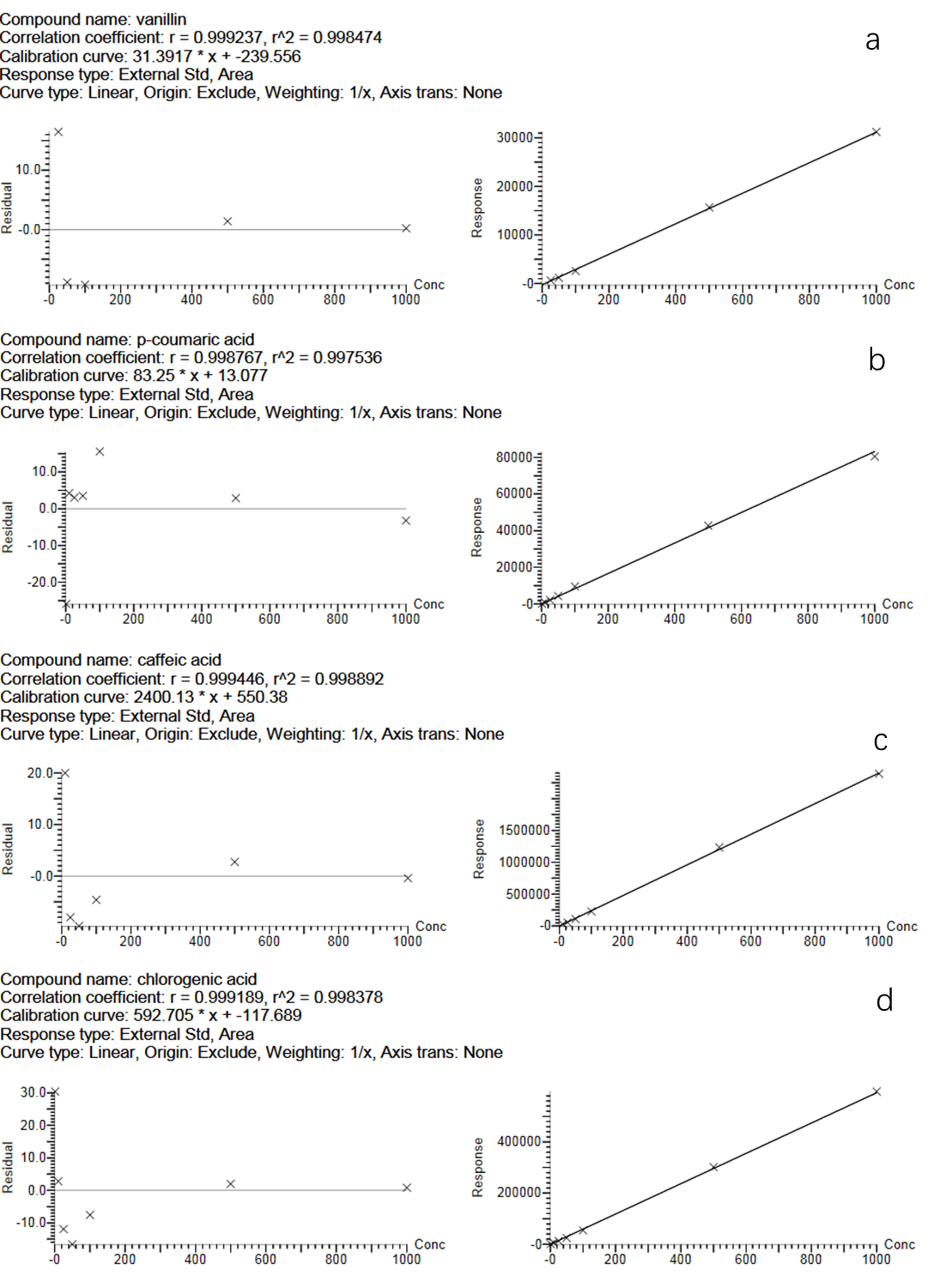
**

**Fig. A.1 Standard curves of four autotoxins prepared by UPLC-MS.** Figure a is the standard curve of vanillin standard, Figure b is the standard curve of *p*-coumaric acid standard, Figure c is the standard curve of caffeic acid standard, Figure d is the standard curve of chlorogenic acid standard. The figure includes the linear range, calibration curves, correlation coefficients and limit of detection of the tested substance.

**2. UPLC-MS analysis and detection results of compositions and contents of autotoxins of *A. artemisiifolia* and *A. trifida***

**Fig. A.2** **UPLC-MS** **chromatograms of four autotoxins in *A. artemisiifolia*.** The figure shows the four autotoxins vanillin (a), *p*-coumaric acid (b), caffeic acid (c), and chlorogenic acid (d) identified from *A. artemisiifolia* plants, and the gray area shows the detected autotoxins.

**Note:** 1. Vanillin (1.86 min);2. *P*-coumaric acid (1.88 min); 3. Caffeic acid (1.13 min); 4. Chlorogenic acid (0.78 min)

**Table A.1** **UPLC-MS parameters of four autotoxins in *A. artemisiifolia***

| Sample Text | Name | Trace | RT | Area | Response | Primar_…_ | Conc. |
| --- | --- | --- | --- | --- | --- | --- | --- |
| 3 | Vanillin | 150.813>135.883 | 1.860 | 701.760 | 701.760 | bb | 30.0 |
| 3 | *P*-coumaric acid | 162.813>119.321 | 1.880 | 871.088 | 871.088 | bb | 10.3 |
| 3 | Caffeic acid | 179.096>135.041 | 1.130 | 86057.234 | 86057.234 | bb | 35.6 |
| 3 | Chlorogenic acid | 352.949>190.946 | 0.780 | 779922.000 | 779922.000 | db | 1316.1 |

**Fig. A.3** **UPLC-MS** **chromatograms of four autotoxins in *A. trifida*.** The figure shows the four autotoxins vanillin (e), *p*-coumaric acid (f), caffeic acid (g), and chlorogenic acid (h) identified from *A. trifida* plants, and the gray area shows the detected autotoxins.

**Note:** 1. Vanillin (1.86 min);2. *P*-coumaric acid (1.88 min); 3. Caffeic acid (1.13 min); 4. Chlorogenic acid (0.78 min)

**Table A.2 UPLC-MS parameters of four** **autotoxins in in *A. trifida***

| Sample Text | Name | Trace | RT | Area | Response | Primar_…_ | Conc. |
| --- | --- | --- | --- | --- | --- | --- | --- |
| 6 | Vanillin | 150.813>135.883 | 1.880 | 671.414 | 671.414 | bb | 29.0 |
| 6 | *P*-coumaric acid | 162.813>119.321 | 1.880 | 2203.958 | 2203.958 | bb | 26.3 |
| 6 | Caffeic acid | 179.096>135.041 | 1.130 | 24410.789 | 24410.789 | bb | 9.9 |
| 6 | Chlorogenic acid | 352.949>190.946 | 0.780 | 222939.516 | 222939.516 | db | 376.3 |
